# Bioactive Compounds Protect Mammalian Reproductive Cells from Xenobiotics and Heat Stress-Induced Oxidative Distress via Nrf2 Signaling Activation: A Narrative Review

**DOI:** 10.3390/antiox13050597

**Published:** 2024-05-13

**Authors:** Muhammad Zahoor Khan, Adnan Khan, Bingjian Huang, Ren Wei, Xiyan Kou, Xinrui Wang, Wenting Chen, Liangliang Li, Muhammad Zahoor, Changfa Wang

**Affiliations:** 1 Liaocheng Research Institute of Donkey High-Efficiency Breeding and Ecological Feeding, Liaocheng University, Liaocheng 522000, China; 2 Genome Analysis Laboratory of the Ministry of Agriculture, Agricultural Genomics Institute at Shenzhen, Chinese Academy of Agricultural Sciences, Shenzhen 511464, China; 3 Department of Molecular Medicine, Institute of Basic Medical Sciences, University of Oslo, Sognsvannsveien, 90372 Oslo, Norway

**Keywords:** reproductive cells, xenobiotics, heat stress, bioactive compounds, oxidative stress, Nrf2 signaling, antioxidant defense

## Abstract

Oxidative stress occurs when there is an imbalance between the production of reactive oxygen species (ROS) and the body’s antioxidant defenses. It poses a significant threat to the physiological function of reproductive cells. Factors such as xenobiotics and heat can worsen this stress, leading to cellular damage and apoptosis, ultimately decreasing reproductive efficiency. The nuclear factor erythroid 2–related factor 2 (Nrf2) signaling pathway plays a crucial role in defending against oxidative stress and protecting reproductive cells via enhancing antioxidant responses. Dysregulation of Nrf2 signaling has been associated with infertility and suboptimal reproductive performance in mammals. Recent advancements in therapeutic interventions have underscored the critical role of Nrf2 in mitigating oxidative damage and restoring the functional integrity of reproductive cells. In this narrative review, we delineate the harmful effects of heat and xenobiotic-induced oxidative stress on reproductive cells and explain how Nrf2 signaling provides protection against these challenges. Recent studies have shown that activating the Nrf2 signaling pathway using various bioactive compounds can ameliorate heat stress and xenobiotic-induced oxidative distress and apoptosis in mammalian reproductive cells. By comprehensively analyzing the existing literature, we propose Nrf2 as a key therapeutic target for mitigating oxidative damage and apoptosis in reproductive cells caused by exposure to xenobiotic exposure and heat stress. Additionally, based on the synthesis of these findings, we discuss the potential of therapies focused on the Nrf2 signaling pathway to improve mammalian reproductive efficiency.

## 1. Introduction

External environmental stressors such as high temperatures and exposure to xenobiotics significantly contribute to the initiation of oxidative stress and apoptosis processes, which have a negative impact on the functionality of reproductive cells [1,2,3,4]. Normally, an organism’s intrinsic antioxidant mechanisms are able to counteract the harmful effects of reactive oxygen species (ROS) overproduction, thus maintaining cellular integrity [5]. However, when there is chronic and excessive ROS generation, oxidative stress occurs, resulting in cellular damage and disruption of normal physiological processes [6]. To mitigate these effects, the use of external antioxidants is recommended to improve cellular antioxidant capacity and influence important biochemical pathways, including the activation of the nuclear factor erythroid 2-related factor 2 (Nrf2) signaling pathway. This intervention aims to protect mammalian reproductive cells from oxidative damage and apoptosis [2,7,8,9].

The Nrf2 protein serves as a vital transcription factor essential for preserving the integrity of redox signaling when cells face oxidative stress [10,11]. As a member of the cap’n’collar basic leucine zipper transcription factor family, Nrf2 plays a vital role in coordinating antioxidant and detoxification responses by upregulating downstream genes [12,13,14]. Under normal conditions, Nrf2 predominantly resides in the cytoplasm, forming a complex with its inhibitory partner, Kelch-like ECH-associated protein 1 (Keap1). However, in the presence of elevated levels of ROS, this complex dissociates, allowing Nrf2 to translocate from the cytoplasm into the nucleus [2,15,16]. Once activated, Nrf2 binds to the antioxidant response element (ARE) sequence, starting the transcription of genes involved in antioxidant defenses to counteract ROS-induced damage [9,17,18,19]. Recent research suggests that p62 competes with Keap1 for binding to the Nrf2 site, disrupting their association, releasing ubiquitinated Nrf2, and subsequently activating the Nrf2–antioxidant systems [20,21].

The role of Nrf2 signaling in safeguarding the reproductive cells/organs against oxidative stress has been extensively studied [22,23,24]. It has been well documented that supplementation of bioactive compounds protects reproductive cells from oxidative stress induced by heat stress and environmental toxicants, via regulation of Nrf2 signaling [25]. Nrf2 has demonstrated protective effects on bovine granulosa cells against H_2_O_2_-induced oxidative stress [26]. Additionally, research by Sun et al. [27] illustrated that supplementation of melatonin safeguarded cryopreserved ovarian tissues from oxidative stress and apoptosis through the Nrf2/HO-1 signaling pathway. They observed an elevation in *Nrf2* levels following melatonin administration, leading to the regulation of antioxidant genes [*glutathione peroxidase* (*GSH-Px*), *catalase* (*CAT*), *superoxide dismutase* (*SOD*), and *heme oxygenase 1* (*HO-1*)] and a reduction in malondialdehyde (MDA) content [27]. Antioxidant responses, including autophagy and *Nrf2* activation, are triggered in response to heat stress-induced apoptosis [9,21]. Alterations in autophagy dynamics play a crucial role in regulating the protective function of the Nrf2 signaling pathway in the testes. This protection involves the suppression of *MDA* levels and the promotion of an antioxidant status that shields the testes from the detrimental effects of heat stress [28,29,30]. Notably, inhibition of *Nrf2* leads to decreased cell viability, increased MDA levels, and Sertoli cell death [11]. Consistently, studies have shown that exposure to heavy metals such as aluminum results in downregulated *Nrf2* expression, increased oxidative stress, and toxicity, negatively impacting male reproductive function [31].

Nrf2 regulates several critical antioxidant genes, such as *CAT*, *heme oxygenase 1* (*HMOX1*), *peroxiredoxin 1* (*PRDX1*), *SOD1*, and *thioredoxin 1* (*TXN1*). These genes collectively enhance antioxidant activity, thereby mitigating oxidative stress in mouse testis cells and safeguarding germ cells and Leydig cells from oxidative damage [30,32]. Recent research has revealed that heat stress-induced ROS overproduction suppresses the expression of antioxidant genes (*SOD*, *CAT*, *quinone oxidoreductase 1* (*NQO1*), and *GSH-Px*) in uterine tissue [33]. In Sertoli cells, heightened ROS levels due to heat stress elevate MDA levels and reduce antioxidant enzyme levels [34]. Additionally, heat stress increases the expression of apoptotic markers such as *Fas*, *FasL*, *caspase 3*, and *caspase 9* in mouse Sertoli cells [34]. Consequently, the Keap1/Nrf2 signaling pathway is significantly associated with the protective effects observed in mouse uterine tissue, characterized by increased levels of antioxidant genes [33].

Moreover, oxidative stress affects various crucial signaling pathways, including the Nrf2/Keap1 signaling axis in the testes [24]. Recent studies emphasize Nrf2’s protective role in shielding mouse Sertoli cells from heat-induced oxidative stress through the Nrf2/Keap1 signaling pathway [11]. Similarly, another investigation demonstrated that Nrf2 significantly reduces caspase 3 levels, consequently decreasing cell death induced by heat stress treatment in Sertoli cells [29]. Under conditions of severe heat stress, heightened expression of Keap1 and Nrf2 facilitates the regulation of genes associated with antioxidants through forming complexes with antioxidant regulated elements (ARE), thus establishing a defensive mechanism against heat stress within bovine endometrial epithelial cells [35]. These findings collectively underscore the critical role of Nrf2 in alleviating oxidative stress and apoptosis in various cellular contexts, particularly under heat stress conditions.

Overall, Nrf2 plays a significant role in regulating the physiology and pathology of reproductive cells via modulating cellular resistance to oxidative stress and apoptosis induced through various factors such as chemicals, environmental toxicants, and heat stress [20]. However, it is notable that several compounds act as both activators and inhibitors of testicular Nrf2. Nrf2 activators potentially hold therapeutic promise in preventing and treating testicular dysfunction, while Nrf2 inhibitors may contribute to dysfunction within testicular components. Activators of Nrf2 confer cellular protection against oxidative damage by stimulating Nrf2-related signaling pathways, facilitating its translocation into the nucleus, and enhancing Nrf2 function and expression, thereby upregulating downstream antioxidant gene expression. Conversely, Nrf2 inhibitors exacerbate oxidative stress by interfering with the Nrf2 signaling pathway. Therefore, this narrative review aims to investigate the impact of xenobiotics and heat stress-induced oxidative distress and apoptosis on the physiology of reproductive cells, while also addressing the protective role of activating the Nrf2 signaling pathway against oxidative stress and apoptosis in mammalian reproductive cells via supplementation of bioactive compounds.

## 2. Methodology

This study’s methodology entailed a comprehensive literature review, primarily focusing on scholarly articles published between 2018 and April 2024. Additionally, select publications dating back to 2013 were also incorporated, specifically those addressing the role of Nrf2 signaling in mitigating oxidative stress and apoptosis induced by heat stress in mammalian reproductive cells. The literature search was conducted using esteemed academic databases, including Google Scholar, Web of Science, X-MOL, and PubMed. The selection of literature was guided by a set of predetermined keywords: “Oxidative Stress”, “Apoptosis”, “Mammalian Reproductive Cells”, “Nrf2 Signaling”, “Xenobiotics”, “Heat Stress”, and “Bioactive Compounds Regulating Nrf2 Signaling”. To ensure the credibility and relevance of the sourced information, only articles published in English and indexed in Science Citation Index (SCI) Journals were considered for this review. In addition, book chapters and articles published in non-English languages were excluded from this review to maintain a focused and high-quality dataset for analysis.

## 3. Administration of Bioactive Compounds Protects Mammalian Reproductive Cells against Xenobiotic and Heat Stress-Induced Oxidative Stress through Nrf2 Signaling Activation 

**Figure 1:**
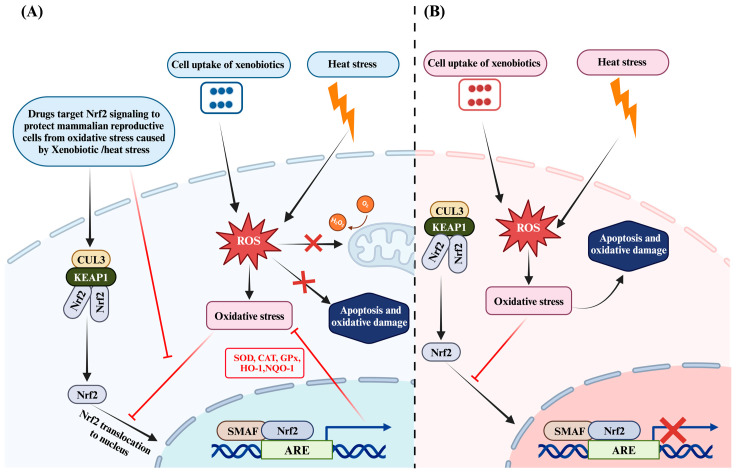
The role of Nrf2 signaling in mitigating heat stress and xenobiotic-induced oxidative distress and apoptosis. (**A**) Exogenous supplementation of bioactive compounds with antioxidant ability activates the Nrf2/KEAP1 signaling pathway. This activation leads to NRF2 translocation to the nucleus and heterodimerization with sMaf proteins. Subsequently, there is binding with ARE to activate antioxidant genes (*SOD*, *CAT*, *NQO1*, *HO1*, and *GPx*). The activation of these antioxidant genes enhances the antioxidant response by suppressing oxidative stress and apoptosis induced via heat stress and xenobiotics. (**B**) Oxidative stress induced by xenobiotics/heat stress increases the level of KEAP1 and inhibits the translocation of NRF2 to the nucleus, leading to oxidative damage and apoptosis of reproductive cells. Note: Blunt arrows (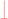) indicate inhibition and sharp arrows (→) indicate stimulation.

The regulation of Nrf2 is intricately managed through its interaction with Keap1. In a state of equilibrium, Keap1 confines Nrf2 within the cytoplasm, maintaining it at minimal levels. This confinement is achieved through the binding of Keap1 to Nrf2 at its C-terminal region, which triggers the ubiquitination of Nrf2. The ubiquitination process, facilitated by the Keap1–Cullin3–RING box protein complex, leads to the subsequent degradation of Nrf2 by the 26S proteasome [29]. During episodes of oxidative distress caused by heat stress or xenobiotics, the increased expression of Keap1 inhibits the translocation of Nrf2 to the nucleus [3,27,33], consequently reducing the antioxidant response (Figure 1B). Conversely, supplementation with bioactive compounds has been observed to downregulate Keap1 expression, resulting in increased Nrf2 levels and subsequent elevation of downstream antioxidant response genes, such as NAD(P)H quinone dehydrogenase 1 (*NQO1*), *HO-1*, *SOD1*, *CAT*, and *GPx* (Figure 1A) [30,36]. Consistently, the pivotal role of Nrf2 in antioxidant defense has been well documented, highlighting its importance in combating oxidative stress and mitigating cellular damage [37,38]. In addition, the therapeutic potential of modulating Nrf2 signaling via administration of bioactive compounds to alleviate oxidative stress has garnered considerable attention in the recent literature [11,14,39,40,41,42,43,44]. Furthermore, the Nrf2 signaling cascade, in conjunction with other protective mechanisms, is crucial in preserving the integrity of mammalian reproductive cells against oxidative stress. This safeguarding is essential for maintaining reproductive health and function.

## 4. Bioactive Compound Supplementation to Combat Xenobiotic-Induced Oxidative Stress and Apoptosis in Reproductive Cells via Activation of the Nrf2 Signaling Pathway 

Xenobiotic agents have been identified as initiators of ROS generation, subsequently inducing oxidative stress [45]. This oxidative milieu has been implicated in impairing the integrity of key reproductive cells, including Sertoli cells, spermatogonial cells, and granulosa cells, potentially underpinning reduced reproductive efficiency and health [46,47,48,49]. The perturbation is manifested through mechanisms such as increased DNA fragmentation in spermatozoa, disruption of mitochondrial membrane lipids in sperm, and compromised functionality of granulosa cells. In response, a spectrum of therapeutic interventions has been explored to fortify reproductive cells against xenobiotic-induced oxidative insult, specifically through the modulation of the Nrf2 signaling cascade [50]. To combat xenobiotic-induced oxidative stress and apoptosis in reproductive cells, several exogenous bioactive compounds with antioxidant properties have been given to animals. These operate via regulating Nrf2 signaling in reproductive cells and consequently ameliorate oxidative damage. Notably, Ji et al. [51] elucidated the ameliorative effects of salidroside on oxidative stress and apoptosis in dihydrotestosterone-challenged human granulosa cells via the AMP-activated protein kinase (AMPK)/Nrf2 pathway, marked by upregulation of *Nrf2*, *HO-1*, and *NQO1*. In a similar way, sulforaphane has been shown to confer protection to bovine granulosa cells against H_2_O_2_-induced oxidative stress through Nrf2 pathway activation, enhancing antioxidant defenses including *SOD*, *CAT*, *NQO1*, and *HO-1*, thereby mitigating oxidative stress and apoptosis [26,52].

Further investigations have revealed that lycopene counteracts dihydrotestosterone-induced oxidative stress in human granulosa cells by activating the Nrf2 signaling pathway [53], while anthocyanins have been reported to safeguard testicular tissue from cadmium-induced oxidative harm through Nrf2 signaling mediation, also revitalizing the activity of key antioxidant enzymes [3]. Targeting the Nrf2/HO-1 axis, carvacrol administration in rats has shown promise in alleviating oxidative stress and apoptosis, evidenced by modulated expression of *Bcl-2*, *Nrf2*, *CAT*, *GPx*, and *HO-1* and reduced MDA and *Bax* levels in testicular tissue [54]. Another study highlighted sitagliptin’s efficacy in attenuating cadmium-induced oxidative stress and toxicity in rats via the Nrf2/HO-1 pathway, resulting in improved testicular health markers [55]. Complementary to these findings, treatments with *Artemisia judaica* extract, ellagic acid, and cardamonin significantly curtailed oxidative stress and apoptosis in diabetic rat testes, underscoring the therapeutic potential of these agents in modulating oxidative balance [56,57,58]. Vitamin D3 has also been recognized for its capacity to mitigate lead-induced oxidative stress and toxicity in rat testes through Nrf2 signaling pathway regulation [59].

In the realm of granulosa cell protection, sulforaphane’s role in enhancing the antioxidant response, thereby shielding the cells from oxidative stress-induced damage, has been reaffirmed [60]. Additionally, vitamin E supplementation has emerged as a viable strategy in bolstering bovine granulosa cell resilience against oxidative stress and apoptosis, facilitated by Nrf2 pathway activation [61]. The deleterious impact of methotrexate on testicular tissue underscores the need for protective agents, with apocynin showing efficacy in safeguarding the testis through Nrf2 signaling pathway activation [62]. Furthermore, the role of deubiquitination in mitigating testicular oxidative stress injury induced by di-n-butylphthalate via the Keap1/Nrf2 signaling pathway has been explored [63]. The emerging concern of diminished ovarian reserve (DOR) in reproductive-aged women, associated with inflammation, has been addressed through studies demonstrating the beneficial effects of moxibustion in modulating the Nrf2/HO-1/nucleotide-binding domain, leucine-rich-containing family, *pyrin domain-containing-3* (*NLRP3*) anti-inflammatory pathway, thereby offering therapeutic insights into DOR management [64]. Collectively, these insights underscore the pivotal role of the Nrf2 signaling pathway in countering the oxidative challenges posed by xenobiotics to mammalian reproductive cells, as encapsulated in Table 1.

## 5. Bioactive Compound Supplementation to Combat Heat Stress Induced Oxidative Stress and Apoptosis in Mammalian Reproductive Cells via Activation of the Nrf2 Signaling Pathway 

Exposure to high temperature has been linked to the activation of cell death and oxidative stress responses within reproductive cells. This phenomenon is supported by a study conducted by Sammad et al. [137], wherein bovine granulosa cells exposed to a thermal stress of 43 °C for 2 h showed a decrease in Nrf2 signaling, resulting in increased apoptosis and oxidative stress markers. Consistently, another study found that heat treatment increased ROS production in granulosa cells with silenced HO-1 and Nrf2 genes [138]. However, granulosa cells with overexpressed HO-1 and Nrf2 genes demonstrated significant resistance, including increased antioxidant response and anti-apoptotic activities [138]. Sertoli cells, which play a vital role in supporting the development of germ cells, rely on normal glucose metabolism for effective spermatogenesis. Melatonin has emerged as a potential therapeutic agent for mitigating the negative effects of heat stress on spermatogenesis. Research by Deng et al. [139] revealed that melatonin reduced heat-induced oxidative stress and apoptotic pathways by activating the KEAP1/Nrf2 signaling axis, thereby enhancing antioxidant defenses. In a parallel finding, He et al. [11] demonstrated the protective role of the Nrf2 signaling pathway against heat stress-induced oxidative challenges in Sertoli cells. Inhibition of the Nrf2 signaling pathway was associated with increased cellular apoptosis, reduced viability, and higher levels of intracellular ROS production. Additionally, melatonin, as reported by Sun et al. [27], upregulated the expression of *heat-shock protein 90* (*HSP90*) through the *melatonin receptor 1B* (*MTNR1B*), which stabilized *hypoxia-inducible factor-1α* (*HIF-1α*). This activation of HIF-1α signaling promoted glycolysis, enhanced the pentose phosphate pathway, and improved cell viability. 

In the domain of uterine physiology, Li et al. [33] observed that heat stress compromised normal uterine function by downregulating Nrf2 expression and its downstream antioxidant genes while upregulating the MDA level. The administration of baicalin significantly improved antioxidant responses and restored normal uterine function. Similarly, Alemu et al. [32] reported downregulated expression of Nrf2 and its target antioxidant genes (*SOD*, *CAT*) in bovine granulosa cells exposed to heat stress, resulting in reduced cell proliferation and increased cell death. Conversely, Li et al. [29] reported an increase in Nrf2 expression after scrotal heat treatment in mouse testes, suggesting a time-dependent response of the Nrf2-antioxidant system to heat stress. Moreover, Li et al. [21] demonstrated that heat stress induced autophagy in mice, activating the Nrf2 signaling pathway as a protective response to oxidative stress, safeguarding testicular tissue from damage. Furthermore, comprehensive research indicates that bioactive compounds given to animals can mitigate oxidative stress and cell death caused by heat stress, while also enhancing the antioxidant response through the regulation of Nrf2 signaling pathways [32,139,140,141,142,143]. 

The collective body of evidence highlights the crucial role of Nrf2 signaling in counteracting oxidative stress caused by heat exposure in mammalian reproductive cells, as summarized in Table 2. These findings emphasize the importance of Nrf2 signaling pathways in the cellular defense mechanism against heat stress-induced reproductive dysfunction.

## 6. Limitations and Future Recommendations

Based on the existing literature, it has been established that most of the evidence presented to date has been derived from in vitro studies and animal models. The absence of clinical trials limits the direct applicability of these findings to reproductive health and, therefore, the therapeutic use of bioactive compounds in clinical settings. Thus, to validate the therapeutic potential of Nrf2 signaling pathway activation in improving reproductive health, clinical trials are needed. These studies should assess the safety, efficacy, and optimal dosing of bioactive compounds in human populations. In addition, the existing review has presented only the protective roles of Nrf2 signaling pathway activation, and it may underrepresent the potential negative effects of prolonged or excessive Nrf2 signaling pathway stimulation, such as possible interference with normal cellular functions or promotion of tumorigenesis in certain contexts. Investigating the long-term effects of chronic Nrf2 signaling pathway mediation on reproductive health is crucial. More detailed mechanistic studies are necessary to better understand how Nrf2 interacts with other cellular pathways under stress conditions. Such studies could lead to the development of more targeted therapies that minimize side effects while enhancing therapeutic efficacy.

## 7. Conclusions

Overall, this review provides compelling evidence that the Nrf2 signaling pathway plays a crucial role in safeguarding mammalian reproductive cells from oxidative stress and apoptosis induced by heat stress and xenobiotic exposure. Through the activation of antioxidant defense genes, the Nrf2 signaling pathway mitigates the harmful effects of heat stress and xenobiotic-induced oxidative stress and apoptosis, thereby preserving the functionality and viability of reproductive cells. Furthermore, the review highlights the pivotal bioactive compounds capable of alleviating oxidative distress caused by heat stress and xenobiotics, safeguarding mammalian reproductive cells from oxidative damage through the activation of the Nrf2 signaling pathway. These findings underscore the importance of Nrf2 in maintaining cellular homeostasis under environmental stressors, highlighting its potential as a therapeutic target for enhancing reproductive health. The intricate regulation of Nrf2 through its interaction with Keap1 and subsequent activation in response to oxidative stress illustrates a sophisticated cellular mechanism for combating cellular damage and maintaining reproductive integrity. Future studies should explore the development of targeted therapies that enhance the Nrf2 signaling pathway, offering new avenues for protecting reproductive health against environmental stressors. Additionally, investigating the long-term effects of Nrf2 modulation on reproductive function could provide deeper insights into its therapeutic potential.

## Figures and Tables

**Table 1:** Summary of studies targeting bioactive compound supplementation to combat xenobiotic-induced oxidative stress and apoptosis in reproductive cells via activation of the Nrf2 signaling pathway.

Xenobiotic-Induced Oxidative Stress/Apoptosis	Therapeutic Agent	Target Pathway	Outcomes	Species	References
Oxidative stress caused by cadmium (Cd)	Anthocyanins (Extract of *Lycium ruthenicum* Murray plant)	Keap1/Nrf2 Signaling Pathway	✧ Mitigated damage to sperm cells, alleviated oxidative stress, and protected testes from toxicity.	Mouse	[3]
Methotrexate-induced oxidative stress	Coenzyme Q10 (CoQ10)	Nrf2/PPAR-γ signaling pathway	✧ Prevented testicular damage and testicular toxicity, primarily via its anti-inflammatory, anti-oxidant, and anti-apoptotic effects.	Rat	[4]
Tripterygium glycoside-induced oxidative stress	Moxibustion	Nrf2/HO-1 signaling pathway	✧ Prevented oligoasthenoteratozoospermia.	Rat	[65]
			✧ Enhances the antioxidant response (increasing T-AOC and T-SOD) and alleviated oxidative stress (decreased MDA) in testes.		
Cisplatin-induced oxidative stress and toxicity	Alpha-pinene (monoterpene)	Nrf2 signaling pathway	✧ Enhanced the level of Nrf2 in testicular tissue.	Rat	[66]
	Syringic acid		✧ Ameliorated oxidative stress and apoptosis and protected testicular tissue from toxicity.		[67]
Type 1 diabetes-induced testicular dysfunction	Icariin	Nrf2 pathway	✧ Enhanced testicular antioxidant capacity by elevating levels of Nrf2.	Mice	[68]
			✧ Improved testicular function.		
Streptozotocin-induced diabetes mellitus, oxidative stress. and apoptosis	Sulbutiamine	Nrf2 signaling pathway	✧ Enhanced antioxidant capacity via elevated expression of *NRF2*, reduced apoptosis via inhibiting levels of Bax and *caspase-3*, and improved the expression of *Bcl-2*.	Mouse	[69]
			✧ Improved testicular weight, testosterone level, sperm number, and motility.		
Oxaliplatin-induced toxicity and oxidative stress	Naringin	Nrf2 signaling pathway	✧ Enhanced the antioxidant response via upregulating the expression of Nrf2 followed by elevated levels of *SOD*, *HO-1*, *NQO1*, and *GPx*.	Rat	[70]
			✧ Inhibited oxaliplatin-induced oxidative stress and toxicity, *caspase-3*, *Bax*, and *Apaf-1* and increased *Bcl2* in OXL-induced testicular toxicity.		
			✧ Protected testicular tissue from the toxic effect of oxaliplatin.		
Sodium benzoate-induced toxicity and oxidative stress	Virgin coconut oil	Nrf2 signaling pathway	✧ Increased the expression of *Nrf2*, *CAT*, and *GPx*.	Rat	[71]
			✧ Relieved oxidative stress and toxicity caused by sodium benzoate.		
			✧ Improved sperm numbers and motility.		
Cyclosporin-induced oxidative stress	Lutein	Nrf2/HO-1 signaling pathway	✧ Promoted antioxidant response (upregulated the expression of *Nrf2*, *CAT*, *GPx*, *SOD*, *HO-1*) and apoptosis (increased the level of *Bcl2*)	Rat	[72]
Diphenyl phosphate (DPhP)-induced apoptosis and reproductive toxicity	Curcumin	Nrf2/P53 signaling pathway	✧ Prevented apoptosis through regulating autophagy via activation of the Nrf2/P53 pathway in mouse spermatocytes.	Mouse	[73]
			✧ Also reduced the risk of reproductive toxicity.		
Cisplatin-induced oxidative stress and toxicity	Arbutin	Nrf2 signaling pathway	✧ Increased expression of *Nrf2*.	Rat	[74]
			✧ Prevented oxidative stress, toxicity, and endoplasmic reticulum stress.		
			✧ Protected ovarian injury caused by cisplatin.		
H_2_O_2_-induced oxidative stress and autophagy	Follicle-stimulating hormone (FSH)	p62/Nrf2 signaling pathway	✧ Protected Sertoli cells from injury via inhibiting oxidative stress and autophagy.	Goat	[75]
Oxidative stress and autophagy caused by Di-(2-ethylhexyl) phthalate (DEHP)	N/A	p62/Keap1/Nrf2 signaling pathway	✧ Protected GCs from oxidative stress and excessive autophagy.	Mouse	[76]
Cadmium-induced oxidative stress and toxicity	Zinc	Nrf2 signaling pathway	✧ Restored the normal function of porcine prepubertal Sertoli cells caused by cadmium toxicity.	pig	[77]
			✧ Enhanced the antioxidant response (upregulated *Nrf2*, *SOD*, *HO-1*, and *GPx* expression) in Sertoli cells.		
Triptolide-induced dysfunction of testicular Sertoli cells, oxidativity, and toxicity	Melatonin	SIRT1/Nrf2 Signaling Pathway	✧ *SIRT1* and *Nrf2* expression levels were enhanced.	Mouse	[78]
			✧ Oxidative stress was relieved and prevented, restoring the normal function of testes.		
Methyl cellosolve-induced oxidative stress	Syringic acid	Nrf2-Keap1-NQO1-HO1 signaling pathway	✧ Enhanced antioxidant response via elevating levels of *Nrf2*, *NQO1*, *HO1*, *SOD*, *CAT*, and *GPx* in testes.	Rat	[79]
			✧ Also decreased the expression of *MDA*		
Torsion–detorsion-induced apoptosis	Pentoxifylline	Nrf2/ARE signaling pathway	✧ Pentoxifylline promoted spermatogenesis.	Mouse	[80]
			✧ Prevented testicular apoptosis by enhancing *Bcl2* and decreasing *caspase-3* and *Bax* expression levels.		
Doxorubicin-induced oxidative stress	Acylated ghrelin	Nrf2/ARE signaling pathway	✧ Enhanced the expression of *Nrf2*, *GSH*, and *SOD*.	Rat	[81]
			✧ Decrease *MDA* level.		
			✧ Elevated antioxidant response.		
			✧ Improved sperm parameters.		
			✧ Prevented doxorubicin-induced testicular damage.		
Streptozotocin-induced testicular damage and oxidative stress	Esculeoside A	Nrf2-signaling pathway	✧ Enhanced *HO-1*, *SOD*, and GSH expression levels.	Rat	[82]
			✧ Decreased the level of *MDA*;		
			✧ Elevated the level of antioxidant response;		
			✧ Improved total sperm count, motility, and survival, reduced head and tail sperm abnormalities, increased circulatory concentrations of follicular stimulating hormone (FSH), testosterone, and luteinizing hormone (LH), and stimulated the testicular expression of several steroidogenic enzymes (StAR, CYP11A1, CYP17A1, 3β-HSD1);		
			✧ Protected testes from oxidative damage caused by streptozotocin.		
2-methoxyethanol-induced testicular oxidative stress	Ferulic acid	Nrf2/Hmox1/NQO1 signaling pathway	✧ Enhanced the expression of *Nrf2*, *GSH*, *SOD*, *Hmox1*, and *NQO1*;	Rat	[83]
			✧ Suppressed the level of *MDA*;		
			✧ Prevented oxidative stress and damage to testes.		
Acrylamide-induced testicular toxicity and oxidative stress	Boron	Nrf2/Keap-1 signaling pathway	✧ Elevated the levels of *Nrf2*, *GSH*, *SOD*, *Keap-1* and reduced the expression of *MDA*;	Rat	[84]
			✧ Promoted the antioxidant response;		
			✧ Protected the testes from toxicity and oxidative damage.		
Lead acetate-induced oxidative stress	Syringic acid	Nrf2 signaling pathway	✧ Improved the expression levels of *SOD*, *GSH*, *GPx*, *CAT*, *Nrf2*, and *NQO1*;	Rat	[85]
			✧ Elevated the level of *Bcl2* and reduced the expression of *Bax* and *caspase-3*		
H_2_O_2_-induced oxidative stress	Kunling Wan (Chinese traditional medicine)	Keap1/Nrf2 signaling pathway	✧ Inhibited oxidative stress and enhanced antioxidant response. Prevented mitochondrial damage;	Mouse	[86]
			✧ Improved oocyte quality.		
H_2_O_2_-induced oxidative stress	Resveratrol	SIRT1/Nrf2/ARE signaling Pathway	✧ Enhance the antioxidant response (increasing T-AOC and T-SOD) and alleviate oxidative stress (decreased MDA).	Rat	[87]
			✧ Suppressed the level of anti-apoptosis protein Bcl-2 and improved the level of pro-apoptosis protein Bax;		
			✧ Prevented ovarian granulosa–lutein cell injury and apoptosis.		
Chlorinated paraffin-induced oxidative stress	Resveratrol	Nrf2 signaling pathway	✧ Prevented testicular toxicity by inhibiting oxidative stress.	Mouse	[88]
3-nitropropionic acid-induced oxidative stress and toxicity	Spermidine	Nrf2/HO-1/GPX4 Signaling Pathway	✧ Prevented apoptosis and oxidative stress and alleviated damage in GCs and ovarian cells.	Mouse and Pig	[89]
Bisphenol AF oxidative stress and apoptosis	Curcumin	Nrf2 signaling pathway	✧ Suppressed intracellular ROS production, discouraged cell apoptosis, downregulated the expression of *Bax* and *cytochrome c*, and upregulated the expression of *Bcl-2*. Reduced the level of *MDA*;	Goat	[90]
			✧ Enhanced the levels of *GSH-Px* and *SOD*;		
			✧ Improved antioxidant response and prevented damage to caprine endometrial epithelial cells.		
Paraquat-induced oxidative stress	Sulforaphane	Keap1/Nrf2 signaling pathway	✧ Inhibited apoptosis via downregulating *Bax* and *caspase-3* expression;	Cow	[91]
			✧ Enhanced antioxidant response via elevating levels of T-SOD and GSH contents;		
			✧ Protected bovine oocytes from cytotoxicity and damage.		
Cadmium-induced oxidative stress and testicular dysfunction	Dapagliflozin	SIRT1/Nrf2/HO-1 signaling pathway	✧ Elevated levels of *GPx*, *Nrf2*, and *SOD* and enhanced antioxidant response;	Rat	[92]
			✧ Decreased expression of *Bax*, increased *Bcl2*, and prevented apoptosis;		
			✧ Improved testicular function.		
Bisphenol F-induced testicular toxicity	Omega-3 fatty acid	Nrf2/NF-kB signaling pathway	✧ Reversed inflammatory changes, enhanced antioxidant response, and prevented testis toxicity.	Rat	[93]
Perfluorooctanesulfonate acid-induced oxidative stress and reproductive injury	1α,25-dihydroxyvitamin D3	Nrf2 signaling pathway	✧ Promoted antioxidant response via elevating levels of *HO-1*, *Nrf2*, *NQO1*, and *SOD2*.	Mouse	[94]
Diethylnitrosamine-induced testicular damage	Sericin and melatonin	Nrf2 signaling pathways	✧ Increased expression of *Nrf2*, *SOD*, *CAT* and *GPx*;	Mouse	[95]
			✧ Enhanced antioxidant capacity;		
			✧ Restored the normal function of testes, which had been impaired by diethylnitrosamine.		
Aflatoxin B1-induced oxidative stress	Lycopene	Nrf2 signaling pathways	✧ Protected testes from aflatoxin B1-induced toxicity and oxidative stress.	Mouse	[96]
H_2_O_2_-induced oxidative stress	Melatonin	Nrf2 signaling pathway	✧ Reduced the level of *MDA* and increased the expression of *Nrf2*, *SOD*, and *Sirt3*;	Mouse	[97]
			✧ Protected Sertoli cells from oxidative stress and prevented infertility.		
Copper-induced toxicity and oxidative stress	Nano-Curcumin	Nrf2/HO-1 signaling pathway	✧ Nano-curcumin and curcumin protected testicular tissue from oxidative injury, enhanced the circulating FSH, LH, and testosterone, and elevated testicular steroidogenesis-linked genes and AR. N-nano-curcumin and curcumin inhibited testicular MDA, NO, NF-κB, iNOS, TNF-α, Bax, and caspase-3 and promoted Bcl-2, Nrf2, and the antioxidants genes including *GSH*, *HO-1*, *SOD*, and *CAT*	Rat	[98]
Cadmium-induced oxidative stress and injury	Zinc	Nrf2 signaling pathway	✧ Elevated the expression of *SOD*, *HO-1*, and *GSHPx* in Sertoli cells;	Pig	[99]
			✧ Protected Sertoli cells from Cd-induced oxidative damage.		
Cadmium-induced oxidative stress	Melatonin	Nrf2 signaling pathway	✧ Antioxidant response was enhanced;	Pig	[100]
			✧ Prevented cytotoxicity of Sertoli cells.		
H_2_O_2_-induced oxidative stress	N-acetyl-cysteine	Nrf2 signaling pathway	✧ Prevented oxidative stress damage to mouse ovaries.	Mouse	[101]
Cisplatin-induced apoptosis and oxidative stress	Melatonin	SIRT1/Nrf2 signaling	✧ Prevented oxidative damage to Leydig and Sertoli cells in the testes.	Mouse	[102]
Zearalenone-induced toxicity and oxidative stress	Betulinic acid	Nrf2-signaling pathway	✧ Protected testes from zearalenone-induced oxidative stress and toxicity.	Mouse	[103]
Zearalenone-induced apoptosis and oxidative stress	Procyanidins	Nrf2 signaling pathway	✧ Enhanced antioxidant response and prevented apoptosis;	Pig	[104]
			✧ Protection of swine testicles from oxidative damage.		
Bisphenol A-induced oxidative stress	Naringenin	Keap1/Nrf2 signaling pathway	✧ Enhanced antioxidant response (enhanced *SOD*, *GPx*, and *CAT* expressions);	Pig	[105]
			✧ Protected swine testes from oxidative damage and cytotoxicity.		
N/A	Curcumin	Nrf2/Keap1 signaling pathway	✧ Enhanced the expression of *Nrf2*, *NQO1*, *HO1*, and *Keap1* genes.	Mouse	[106]
			✧ Prevent cryptorchidism complications		
Fumonisin-induced oxidative stress	N/A	Nrf2 signaling pathway	✧ Increased ROS level, reduced expression of *MDA*, disrupted the Keap1-Nrf2 pathway, and compromised the antioxidant system of the testes.	Mouse	[107]
Cadmium-induced oxidative stress and toxicity	Vitamin E	Nrf2 signaling pathway	✧ Reduced expression of *MDA* and enhanced activities of *T-AOC*, *GSH*, *CAT*, *SOD*, and *GSH-Px*;	Rat	[108]
			✧ Enhanced antioxidant response and prevented oxidative damage to testes;		
			✧ Elevated rate of normal sperm, increased sperm count, motility, and viability.		
Triptolide-induced oxidative stress and apoptosis	Hyperoside	Keap1-Nrf2 signaling pathway	✧ Upregulated the expressions of *Nrf2*, *SOD* and *GPx*, and decreased caspase-3;	Mouse	[109]
			✧ Prevented testicular atrophy and injury.		
Zearalenone-induced oxidative stress and impaired steroidogenesis	Zingerone	Nrf2 signaling pathway	✧ Enhanced antioxidant response by upregulating *Nrf2* expression in Leydig cells.	Mouse	[110]
			✧ Improved steroidogenesis.		
Testicular torsion/detorsion-induced injury enhancing inflammation, suppression of Nrf2 siganling, and oxidative stress	Idebenone	Nrf2 signaling pathway	✧ Decreased the levels of *MDA* and *caspase-3* and enhanced the expression of *Nrf2*;	Mouse	[111]
			✧ Relieved apoptosis and inflammation and improved antioxidant response;		
			✧ Protected testes from testicular torsion injury.		
Fructose–streptozotocin-impaired steroidogenesis and spermatogenesis	Caffeic acid	Nrf2 signaling pathway	✧ Enhanced *Nrf2* expression and restored the normal process of steroidogenesis and spermatogenesis in testes of mice.	Mouse	[112]
LPS-induced oxidative stress in bovine endometrial cells	Selenium	Nrf2/HO-1 signaling pathway	✧ Enhanced the levels of *Nrf2* and *GPx*;	Cow	[113]
			✧ Relieved oxidative stress and prevented endometritis.		
Zearalenone-induced oxidative damage	Lycopene	Nrf2 signaling pathway	✧ Enhanced the antioxidant response genes (increased expressions of *GPx*, *Nrf2*, *HO1*, and *SOD*).	Pig	[114]
N/A	Qiangjing Tablets	Nrf2 signaling pathway	✧ Enhanced sperm motility, concentration, and viability, which was linked significantly increased levels of HO-1, Keap1, P-Nrf2, estradiol, and testosterone, along with increasing the activity of *SOD*, *GSH-Px*, and *GSH* and suppressing *MDA* content, luteinizing hormone, and vimentin levels.	Mouse	[115]
			✧ Protected spermatogenic cells to upregulate male sex hormone, improved sperm quality and reproductive function in asthenozoospermia rats via activating the Keap/Nrf2 signaling pathway.		
N/A	Chitooligosaccharide-zinc	SESN2/Nrf2 signaling pathway	✧ Prevented premature ovarian failure and enhanced ovarian and follicular development via activation of the SESN2/Nrf2 signaling pathway;	Mouse	[116]
			✧ *SOD*, *Nrf2*, and *SESN2* expression levels were upregulated following improved antioxidant response.		
Lead-induced oxidative stress and apoptosis	Luteolin	Nrf2/HO-1 signaling pathway	✧ Prevented testicular tissue injury by relieving apoptosis (decreased Bax and caspase 3) and enhanced antioxidant response via elevated expressions of *GPx*, *Nrf2*, *HO-1*, *NQO1* in testicular tissue.	Rat	[117]
La_2_O_3_ nanoparticle-induced oxidative stress apoptosis and toxicity	N/A	Nrf2/HO-1 signaling pathway	✧ Prevented the translocation of Nrf2 to the nucleus;	Mouse	[118]
			✧ Enhanced apoptosis and oxidative and testicular tissue injury.		
Cadmium-induced oxidative stress and apoptosis	Ferulic acid	Nrf2 signaling pathway	✧ Upregulated the expressions of *Nrf2*, *SOD*, *CAT*, and *GPx*;	Rat	[119]
			✧ Improved antioxidant response and protected testicular injury.		
Cisplatin-induced oxidative stress and apoptosis	Tadalafil	Nrf2/HO-1 signaling pathway	✧ Promoted the antioxidant response (*Nrf2* and *HO-1*) and inhibited apoptosis (decreased *Bax* and enhanced *Bcl2* expression).	Rat	[120]
			✧ Prevented testicular oxidative damage and toxicity in rats.		
N/A	Quercetin	Nrf2 signaling pathway	✧ Enhanced antioxidant response via upregulating the expression of *Nrf2*, *NQO1*, *PRDX1*, *CAT*, and *SOD1*;	Cow	[121]
			✧ Protected preimplantation embryos against oxidative stress and improved embryo viability via activation of the Nrf2 signaling pathway.		
Aluminium chloride-induced oxidative stress and apoptosis	Tyrosol	Nrf2/HO-1 signaling pathway	✧ Protected testicular toxicity and oxidative damage and improved sperm motility by upregulating *GSH*, *CAT*, *Nrf2*, *HO-1*, and *bcl-2* expression and downregulating *caspase-3* and *MDA* levels.	Rat	[122]
Zearalenone-induced apoptosis and oxidative stress.	Curcumin	Nrf2 signaling pathway	✧ Protected Leydig cells from oxidative stress and apoptosis via regulation of the Nrf2 signaling pathway;	Mouse	[123]
			✧ Enhanced the antioxidant response (Increased *Nrf2*, *HO-1*, *SOD*, *GSH*, and GSH-Px expressions, and reduced *MDA* level) and relieved apoptosis (enhanced *Bcl-2* and reduced *Bax* levels).		
Cadmium-induced oxidative stress and apoptosis	Sulforaphane	Nrf2/ARE signaling pathway	✧ Protected Leydig cells from oxidative stress and apoptosis via regulation of the Nrf2 signaling pathway.	Mouse	[124]
			✧ Enhanced the antioxidant response (*Nrf2*, *GSH-Px*, *HO-1*, *γ-GCS*, and *NQO1* expression, reduced *MDA* level) and relieved apoptosis;		
			✧ Protected testicular tissue toxicity.		
Doxorubicin-induced oxidative stress and toxicity	N/A	Nrf2 signaling pathway	✧ Suppressed the level of *Nrf2*, enhanced apoptosis (decreased *caspase 3* and *Bcl2* levels) and oxidative stress;	Mouse	[125]
			✧ Caused testicular tissue toxicity and injury.		
di(2-ethylhexyl) phthalate (DEHP)-induced oxidative stress and Leydig cell damage	Lycopene	Nrf2 signaling pathway	✧ Elevated level of *Nrf2* and its antioxidant linked genes (*HO-1*, *NQO1*);	Mouse	[126]
			✧ Prevented oxidative stress and Leydig cell injury in mice.		
Fluoride-induced testicular apoptosis	N-acetylcysteine	Nrf2 signaling pathway	✧ Improved the antioxidant response and alleviated apoptosis via activation of the Nrf2 pathway;	Mouse	[127]
			✧ Protected testis tissue from oxidative damage.		
Cadmium-induced testicular injury and oxidative stress	Curcumin	Nrf2/ARE signaling pathway	✧ Upregulated the expression levels of *T-SOD*, *GSH-Px*, *GSH*, *Nrf2*, and *γ-GCS* and reduced the level of *MDA* in testicular tissue;	Mouse	[128]
			✧ Prevented testicular injury.		
Cadmium-induced testicular injury and oxidative stress	Piceatannol	Nrf2 signaling pathway	✧ Piceatannol inhibited oxidative stress via upregulation of antioxidant genes (*Nrf2*, *HO1*, *γGCS*, *GPx*, and *NQO1*).	Rat	[129]
Cd-induced oxidative stress and apoptosis	Sulforaphane	Nrf2/ARE signaling pathway	✧ Upregulated the expression of antioxidant linked genes (*Nrf2*, *T-SOD*, *HO-1*, *NQO1*, *GSH-Px*, and *γ-GCS*);	Mouse	[130]
	proanthocyanidins		✧ Protective against oxidative damage and apoptosis caused by Cd in Sertoli cells.		[131]
Dihydrotestosterone-induced oxidative stress	Salidroside	Nrf2 signaling pathway	✧ Suppressed apoptosis and oxidative stress; ✧ Protected human granulosa cell from oxidative stress.		[51]
H_2_O_2_-induced oxidative damage and ferroptosis	Pterostilbene	Nrf2/HO-1 signaling pathway	✧ Inhibited oxidative damage and ferroptosis in human ovarian granulosa cells via Nrf2/HO-1 signaling pathway.		[132]
H_2_O_2_-induced oxidative stress	Sulforaphane	AMPK/AKT/NRF2 signaling pathway	✧ Suppressed apoptosis and oxidative stress; ✧ Increased the expression of *AMPK*, *AKT*, and *NRF2*; ✧ Protected human granulosa–lutein cells from the injury of oxidative stress.		[133]
H_2_O_2_-induced oxidative stress	Morroniside	Nrf2 signaling pathway	✧ Improved antioxidant response (increased expression of *Nrf2*, *SOD*, and *NQO1* and decreased level of *MDA*); ✧ Prevented apoptosis; ✧ Improved quality of oocytes; ✧ Protected ovarian granulosa cells from oxidative stress and apoptosis.	Human	[134]
Busulfan-induced oxidative stress and apoptosis	Astaxanthin	Nrf2/HO-1 signaling pathway	✧ Relieved oxidative stress by enhancing antioxidant response (increased expression of *Nrf2*, *HO-1*, and *SOD*) and reduced apoptosis genes (decreased levels of *CASP9*, *CASP3*, *Bax* and suppressed the *BCL2* content) in human spermatogonial stem cells.		[135]
Polycystic ovary syndrome (PCOS)-induced oxidative stress	Sulforaphane	Nrf2 signaling pathway	✧ Protected granulosa cells from PCOS-induced oxidative stress; ✧ Activated Nrf2 signaling to enhance the antioxidant response.		[136]

*Total antioxidant capacity* (*T-AOC*)*; total superoxide dismutase* (*T-SOD*)*; malondialdehyde* (*MDA*)*; granulosa cells* (*GCs*)*; nuclear factor E2-related factor 2* (*Nrf2*)*/antioxidant response element* (*ARE*)*; heme oxygenase-1* (*HO-1*)*; glutathione peroxidase* (*GSH-Px*)*; quinone oxidoreductase 1* (*NQO1*)*; sirtuin 3* (*Sirt3*)*; androgen receptor* (*AR*)*; kelch-like ECH-associated protein 1* (*KEAP1*)*; peroxiredoxin 1* (*PRDX1*)*; γ-glutamylcysteine synthetase* (*γ-GCS*)*; Tumour Necrosis Factor alpha* (*TNF-α*)*; steroidogenic acute regulatory protein* (*StAR*)*; cytochrome P450 family 11 subfamily A member 1* (*CYP11A1*)*; human 3 beta-hydroxysteroid dehydrogenase deficiency* (*3β-HSD1*)*; nitric oxide* (*NO*)*; inducible nitric oxide synthase* (*iNOS*)*, nuclear factor-κB* (*NF-κB*).

**Table 2:** Summary of studies investigation supplementation with bioactive compounds to combat heat stress-induced oxidative distress and apoptosis in mammalian reproductive cells via activation of the Nrf2 signaling pathway.

Causative Agent of Oxidative Stress/Apoptosis	Therapeutic Agent	Target Pathway	Outcomes	Species	References
Heat stress-induced oxidative stress	N/A	Nrf2 signaling pathway	✧ Treatment with 41 °C for 24 h significantly downregulated the levels of *CAT*, *SOD*, and *Nrf2* gene expression; ✧ Upregulated the level apoptosis by regulating *BAX* and *caspase-3* in bovine granulosa cells; ✧ Heat-induced oxidative stress compromised cell proliferation and apoptosis.	Cow	[32]
Heat stress-induced oxidative stress and uterine injuries	Baicalin	Keap1/Nrf2 signaling pathway	✧ Enhanced the levels of antioxidant genes (*SOD*, *CAT*, *GSH-Px*) and decreased the level of *MDA*; ✧ Inhibited the expression of *caspase-3* and *caspase-9* in mouse uterine cells; ✧ Protected mouse uterine cells from heat stress-induced oxidative injures and apoptosis.	Mouse	[33]
Heat stress-induced oxidative stress in bovine endometrial cells	N/A	Keap1/Nrf2 signaling pathway	✧ Activated *Nrf2* further regulated antioxidant-linked genes to balance the oxidative stress.	Cow	[35]
Heat stress-induced oxidative stress and apoptosis	Melatonin	Keap1/Nrf2 signaling pathway	✧ Alleviated oxidative stress and apoptosis via activating Keap1/Nrf2 signaling in Sertoli cells.	Mouse	[139]
Heat stress-induced oxidative stress	Selenium	Nrf2 signaling pathway	✧ Upregulated *GPX-4*, *SOD*, and *CAT* and downregulated *MDA*.	Cow	[140]
Heat stress-induced oxidative stress and toxicity	*Asparagus officinalis* stem	Nrf2 signaling pathway	✧ Enhanced the levels of *HSP70*, *Nrf2*, *Keap1*, and *HSF1* in bovine cumulus–granulosa cells; ✧ Protected bovine cumulus–granulosa cells from oxidative damage and toxicity.	Cow	[141]
Scrotal heat-mediated damage and infertility	Camel whey protein	Nrf2 signaling pathway	✧ Camel milk significantly upregulated the expression of *Nrf2* and *BCL2*, which were downregulated in Leydig cells through scrotal heating.	Mouse	[142]
Heat stress-induced inflammation and oxidative stress	Tender coconut water	NF-κB/Nrf2 signaling pathway	✧ Prevented testicular damage and enhanced the antioxidant response.	Mouse	[143]

## Data Availability

All the data are available in the manuscript.

## References

[1] Zhang S.X., Wang D.L., Qi J.J., Yang Y.W., Sun H., Sun B.X., Liang S. (2024). Chlorogenic Acid Ameliorates the Heat Stress-Induced Impairment of Porcine Sertoli Cells by Suppressing Oxidative Stress and Apoptosis. Theriogenology.

[2] Khan M.Z., Khan A., Chen W., Chai W., Wang C. (2024). Advancements in Genetic Biomarkers and Exogenous Antioxidant Supplementation for Safeguarding Mammalian Cells against Heat-Induced Oxidative Stress and Apoptosis. Antioxidants.

[3] Dong M., Lu J., Xue H., Lou Y., Li S., Liu T., Ding Z., Chen X. (2024). Anthocyanins from *Lycium ruthenicum* Murray Mitigate Cadmium-Induced Oxidative Stress and Testicular Toxicity by Activating the Keap1/Nrf2 Signaling Pathway. Pharmaceuticals.

[4] Arafa E.S., Hassanein E.H., Ibrahim N.A., Buabeid M.A., Mohamed W.R. (2024). Involvement of Nrf2-PPAR-γ Signaling in Coenzyme Q10 Protecting Effect Against Methotrexate-Induced Testicular Oxidative Damage. Int. Immunopharmacol..

[5] Ribeiro J.C., Braga P.C., Martins A.D., Silva B.M., Alves M.G., Oliveira P.F. (2021). Antioxidants Present in Reproductive Tract Fluids and Their Relevance for Fertility. Antioxidants.

[6] Khan A., Dou J., Wang Y., Jiang X., Khan M.Z., Luo H., Usman T., Zhu H. (2020). Evaluation of Heat Stress Effects on Cellular and Transcriptional Adaptation of Bovine Granulosa Cells. J. Anim. Sci. Biotechnol..

[7] Saini S., Selokar N.L., Singh M.K. (2024). Curcumin Supplementation Ameliorates Heat Stress and Affects Early Embryonic Development. Anim. Reprod. Update.

[8] Samir H., Samir M., Radwan F., Mandour A.S., El-Sherbiny H.R., Ahmed A.E., Al Syaad K.M., Al-Saeed F.A., Watanabe G. (2024). Effect of Pre-Treatment of Melatonin on Superovulation Response, Circulatory Hormones, and miRNAs in Goats During Environmental Heat Stress Conditions. Vet. Res. Commun..

[9] Signorini C., Saso L., Ghareghomi S., Telkoparan-Akillilar P., Collodel G., Moretti E. (2024). Redox Homeostasis and Nrf2-Regulated Mechanisms Are Relevant to Male Infertility. Antioxidants.

[10] Bonay M. (2024). Molecular Targets of Oxidative Stress: Focus on the Nrf2 Signaling Pathway in Health and Disease. Antioxidants.

[11] He C., Sun J., Yang D., He W., Wang J., Qin D., Zhang H., Cai H., Liu Y., Li N. (2022). Nrf2 activation mediates the protection of mouse Sertoli Cells damage under acute heat stress conditions. Theriogenology.

[12] Cui W., Li B., Bai Y., Miao X., Chen Q., Sun W., Tan Y., Luo P., Zhang C., Zheng S. (2013). Potential role for Nrf2 activation in the therapeutic effect of MG132 on diabetic nephropathy in OVE26 diabetic mice. Am. J. Physiol. Endocrinol. Metab..

[13] Díaz M., Valdés-Baizabal C., de Pablo D.P., Marin R. (2024). Age-Dependent Changes in Nrf2/Keap1 and Target Antioxidant Protein Expression Correlate to Lipoxidative Adducts, and Are Modulated by Dietary N-3 LCPUFA in the Hippocampus of Mice. Antioxidants.

[14] El Kebbaj R., Bouchab H., Tahri-Joutey M., Rabbaa S., Limami Y., Nasser B., Egbujor M.C., Tucci P., Andreoletti P., Saso L. (2024). The Potential Role of Major Argan Oil Compounds as Nrf2 Regulators and Their Antioxidant Effects. Antioxidants.

[15] Suzuki T., Takahashi J., Yamamoto M. (2023). Molecular Basis of the KEAP1-NRF2 Signaling Pathway. Mol. Cells.

[16] Ucar B.I., Ucar G., Saha S., Buttari B., Profumo E., Saso L. (2021). Pharmacological Protection Against Ischemia-Reperfusion Injury by Regulating the Nrf2-Keap1-ARE Signaling Pathway. Antioxidants.

[17] Xi C., Palani C., Takezaki M., Shi H., Horuzsko A., Pace B.S., Zhu X. (2024). Simvastatin-Mediated Nrf2 Activation Induces Fetal Hemoglobin and Antioxidant Enzyme Expression to Ameliorate the Phenotype of Sickle Cell Disease. Antioxidants.

[18] Chakkittukandiyil A., Sajini D.V., Karuppaiah A., Selvaraj D. (2022). The Principal Molecular Mechanisms Behind the Activation of Keap1/Nrf2/ARE Pathway Leading to Neuroprotective Action in Parkinson’s Disease. Neurochem. Int..

[19] Ulasov A.V., Rosenkranz A.A., Georgiev G.P., Sobolev A.S. (2022). Nrf2/Keap1/ARE Signaling: Towards Specific Regulation. Life Sci..

[20] Rotimi D.E., Ojo O.A., Olaolu T.D., Adeyemi O.S. (2022). Exploring Nrf2 as a Therapeutic Target in Testicular Dysfunction. Cell Tissue Res..

[21] Li Z., Li Y., Zhou X., Dai P., Li C. (2018). Autophagy Involved in the Activation of the Nrf2-Antioxidant System in Testes of Heat-Exposed Mice. J. Thermal Biol..

[22] Ding X., Ge B., Wang M., Zhou H., Sang R., Yu Y., Xu L., Zhang X. (2020). Inonotus obliquus Polysaccharide Ameliorates Impaired Reproductive Function Caused by Toxoplasma gondii Infection in Male Mice via Regulating Nrf2-PI3K/AKT Pathway. Int. J. Biol. Macromol..

[23] Farkhondeh T., Folgado S.L., Pourbagher-Shahri A.M., Ashrafizadeh M., Samarghandian S. (2020). The Therapeutic Effect of Resveratrol: Focusing on the Nrf2 Signaling Pathway. Biomed. Pharmacother..

[24] Feng J., He Y., Shen Y., Zhang G., Ma S., Zhao X., Zhang Y. (2020). Protective Effects of Nuclear Factor Erythroid 2-Related Factor on Oxidative Stress and Apoptosis in the Testis of Mice Before Adulthood. Theriogenology.

[25] Vašková J., Klepcová Z., Špaková I., Urdzík P., Štofilová J., Bertková I., Kľoc M., Rabajdová M. (2023). The Importance of Natural Antioxidants in Female Reproduction. Antioxidants.

[26] Taqi M.O., Saeed-Zidane M., Gebremedhn S., Salilew-Wondim D., Tholen E., Neuhoff C., Hoelker M., Schellander K., Tesfaye D. (2021). NRF2-Mediated Signaling is a Master Regulator of Transcription Factors in Bovine Granulosa Cells Under Oxidative Stress Condition. Cell Tissue Res..

[27] Sun T.C., Liu X.C., Yang S.H., Song L.L., Zhou S.J., Deng S.L., Tian L., Cheng L.Y. (2020). Melatonin Inhibits Oxidative Stress and Apoptosis in Cryopreserved Ovarian Tissues via Nrf2/HO-1 Signaling Pathway. Front. Mol. Biosci..

[28] Li Y., Cao Y., Wang F., Li C. (2014). Scrotal heat induced the Nrf2-driven antioxidant response during oxidative stress and apoptosis in the mouse testis. Acta Histochem..

[29] Li Y., Cao Y., Wang F., Pu S., Zhang Y., Li C. (2014). Tert-butylhydroquinone attenuates scrotal heat-induced dam-age by regulating Nrf2-antioxidant system in the mouse testis. Gen. Comp. Endocrinol..

[30] Li Y., Huang Y., Piao Y., Nagaoka K., Watanabe G., Taya K., Li C.M. (2013). Protective effects of nuclear factor erythroid 2-related factor 2 on whole body heat stress-induced oxidative damage in the mouse testis. Reprod. Biol. Endocrinol..

[31] Ali F.E., Badran K.S., Baraka M.A., Althagafy H.S., Hassanein E.H. (2024). Mechanism and impact of heavy met-al-aluminum (Al) toxicity on male reproduction: Therapeutic approaches with some phytochemicals. Life Sci..

[32] Alemu T.W., Pandey H.O., Wondim D.S., Gebremedhn S., Neuhof C., Tholen E., Holker M., Schellander K., Tesfaye D. (2018). Oxidative and endoplasmic reticulum stress defense mechanisms of bovine granulosa cells exposed to heat stress. Theriogenology.

[33] Li H., Cong X., Yu W., Jiang Z., Fu K., Cao R., Tian W., Feng Y. (2022). Baicalin inhibits oxidative injures of mouse uterine tissue induced by acute heat stress through activating the Keap1/Nrf2 signaling pathway. Res. Vet. Sci..

[34] Sui J., Feng Y., Li H., Cao R., Tian W., Jiang Z. (2019). Baicalin protects mouse testis from injury induced by heat stress. J. Therm. Biol..

[35] Murata H., Kunii H., Kusama K., Sakurai T., Bai H., Kawahara M., Takahashi M. (2021). Heat stress induces oxidative stress and activates the KEAP1-NFE2L2-ARE pathway in bovine endometrial epithelial cells. Biol. Reprod..

[36] Hammad M., Raftari M., Cesário R., Salma R., Godoy P., Emami S.N., Haghdoost S. (2023). Roles of oxidative stress and Nrf2 signaling in pathogenic and non-pathogenic cells: A possible general mechanism of resistance to therapy. Antioxidants.

[37] Yin C., Bi Q., Chen W., Wang C., Castiglioni B., Li Y., Sun W., Pi Y., Bontempo V., Li X. (2024). Fucoidan Supplementation Improves Antioxidant Capacity via Regulating the Keap1/Nrf2 Signaling Pathway and Mitochondrial Function in Low-Weaning Weight Piglets. Antioxidants.

[38] Ngo V., Duennwald M.L. (2022). Nrf2 and oxidative stress: A general overview of mechanisms and implications in human disease. Antioxidants.

[39] Glanzner W.G., da Silva Sousa L.R., Gutierrez K., de Macedo M.P., Currin L., Perecin F., Bordignon V. (2024). NRF2 attenuation aggravates detrimental consequences of metabolic stress on cultured porcine parthenote embryos. Sci. Rep..

[40] Li Y., Cai L., Bi Q., Sun W., Pi Y., Jiang X., Li X. (2024). Genistein Alleviates Intestinal Oxidative Stress by Activating the Nrf2 Signaling Pathway in IPEC-J2 Cells. Vet. Sci..

[41] Xiong L., Azad M.A., Liu Y., Zhang W., Zhu Q., Hu C., You J., Kong X. (2024). Intrauterine Growth Restriction Affects Colonic Barrier Function via Regulating the Nrf2/Keap1 and TLR4-NF-κB/ERK Pathways and Altering Colonic Microbiome and Metabolome Homeostasis in Growing–Finishing Pigs. Antioxidants.

[42] Zheng S.L., Wang Y.M., Chi C.F., Wang B. (2024). Chemical Characterization of Honeysuckle Polyphenols and Their Alleviating Function on Ultraviolet B-Damaged HaCaT Cells by Modulating the Nrf2/NF-κB Signaling Pathways. Antioxidants.

[43] Yuan M., Fu H., Mo Q., Wang S., Wang C., Wang D., Zhang J., Li M. (2024). Protective Mechanism of Rosa roxburghii Tratt Fermentation Broth against Ultraviolet-A-Induced Photoaging of Human Embryonic Skin Fibroblasts. Antioxidants.

[44] Thiruvengadam M., Venkidasamy B., Subramanian U., Samynathan R., Ali Shariati M., Rebezov M., Girish S., Thangavel S., Dhanapal A.R., Fedoseeva N. (2021). Bioactive Compounds in Oxidative Stress-Mediated Diseases: Targeting the NRF2/ARE Signaling Pathway and Epigenetic Regulation. Antioxidants.

[45] Buha A., Baralić K., Djukic-Cosic D., Bulat Z., Tinkov A., Panieri E., Saso L. (2021). The role of toxic metals and metalloids in Nrf2 signaling. Antioxidants.

[46] El-Din M.A., Ghareeb A.E., El-Garawani I.M., El-Rahman H.A. (2023). Induction of apoptosis, oxidative stress, hormonal, and histological alterations in the reproductive system of thiamethoxam-exposed female rats. Environ. Sci. Pollut. Res..

[47] Deluao J.C., Winstanley Y., Robker R.L., Pacella-Ince L., Gonzalez M.B., McPherson N.O. (2022). Oxidative stress and reproductive function: Reactive oxygen species in the mammalian pre-implantation embryo. Reproduction.

[48] Jiang X., Xing X., Zhang Y., Zhang C., Wu Y., Chen Y., Meng R., Jia H., Cheng Y., Zhang Y. (2021). Lead exposure activates the Nrf2/Keap1 pathway, aggravates oxidative stress, and induces reproductive damage in female mice. Ecotoxicol. Environ. Saf..

[49] Meli R., Monnolo A., Annunziata C., Pirozzi C., Ferrante M.C. (2020). Oxidative stress and BPA toxicity: An antioxidant approach for male and female reproductive dysfunction. Antioxidants.

[50] Chung J.Y., Chen H., Zirkin B. (2021). Sirt1 and Nrf2: Regulation of Leydig cell oxidant/antioxidant intracellular environment and steroid formation. Biol. Reprod..

[51] Ji R., Jia F.Y., Chen X., Wang Z.H., Jin W.Y., Yang J. (2022). Salidroside alleviates oxidative stress and apoptosis via AMPK/Nrf2 pathway in DHT-induced human granulosa cell line KGN. Arch. Biochem. Biophys..

[52] Sohel M.M., Amin A., Prastowo S., Linares-Otoya L., Hoelker M., Schellander K., Tesfaye D. (2018). Sulforaphane protects granulosa cells against oxidative stress via activation of NRF2-ARE pathway. Cell Tissue Res..

[53] Chen Y., Zhao M., Li X., Liu Y., Shang Y. (2024). Lycopene mitigates DHT-induced apoptosis and oxidative stress in human granulosa cell line KGN by regulating the Nrf2 pathway. Mol. Cell Toxicol..

[54] Arkali G., Aksakal M., Kaya Ş.Ö. (2021). Protective effects of carvacrol against diabetes-induced reproductive damage in male rats: Modulation of Nrf2/HO-1 signalling pathway and inhibition of Nf-kB-mediated testicular apoptosis and inflammation. Andrologia.

[55] Arab H.H., Gad A.M., Reda E., Yahia R., Eid A.H. (2021). Activation of autophagy by sitagliptin attenuates cadmium-induced testicular impairment in rats: Targeting AMPK/mTOR and Nrf2/HO-1 pathways. Life Sci..

[56] ALTamimi J.Z., AlFaris N.A., Aljabryn D.H., Alagal R.I., Alshammari G.M., Aldera H., Alqahtani S., Yahya M.A. (2021). Ellagic acid improved diabetes mellitus-induced testicular damage and sperm abnormalities by activation of Nrf2. Saudi J. Biol. Sci..

[57] Saeedan A.S., Soliman G.A., Abdel-Rahman R.F., Abd-Elsalam R.M., Ogaly H.A., Foudah A.I., Abdel-Kader M.S. (2021). Artemisia judaica L. diminishes diabetes-induced reproductive dysfunction in male rats via activation of Nrf2/HO-1-mediated antioxidant responses. Saudi J. Biol. Sci..

[58] Samir S.M., Elalfy M., El Nashar E.M., Alghamdi M.A., Hamza E., Serria M.S., Elhadidy M.G. (2021). Cardamonin exerts a protective effect against autophagy and apoptosis in the testicles of diabetic male rats through the expression of Nrf2 via p62-mediated Keap-1 degradation. Korean J. Physiol. Pharmacol..

[59] Abbaszadeh S., Yadegari P., Imani A., Taghdir M. (2021). Vitamin D3 protects against lead-induced testicular toxicity by modulating Nrf2 and NF-κB genes expression in rat. Reprod. Toxicol..

[60] Esfandyari S., Aleyasin A., Noroozi Z., Taheri M., Khodarahmian M., Eslami M., Rashidi Z., Amidi F. (2021). The protective effect of sulforaphane against oxidative stress through activation of NRF2/ARE pathway in human granulosa cells. Cell J..

[61] Wang M., Li Y., Gao Y., Li Q., Cao Y., Shen Y., Chen P., Yan J., Li J. (2021). Vitamin E regulates bovine granulosa cell apoptosis via NRF2-mediated defence mechanism by activating PI3K/AKT and ERK1/2 signalling pathways. Reprod. Domest. Anim..

[62] Sayed A.M., Hassanein E.H., Ali F.E., Omar Z.M., Rashwan E.K., Mohammedsaleh Z.M., Abd El-Ghafar O.A. (2021). Regulation of Keap-1/Nrf2/AKT and iNOS/NF-κB/TLR4 signals by apocynin abrogated methotrexate-induced testicular toxicity: Mechanistic insights and computational pharmacological analysis. Life Sci..

[63] Zhang L., Gao X., Qin Z., Shi X., Xu K., Wang S., Tang M., Wang W., Gao S., Zuo L. (2021). USP15 Participates in DBP-Induced Testicular Oxidative Stress Injury through Regulating the Keap1/Nrf2 Signaling Pathway. Sci. Total Environ..

[64] Lu G., Wang Q., Xie Z.J., Liang S.J., Li H.X., Shi L., Li Q., Shen J., Cheng J., Shen M.H. (2021). Moxibustion Ameliorates Ovarian Reserve in Rats by Mediating Nrf2/HO-1/NLRP3 Anti-Inflammatory Pathway. Evid. Based Complement. Alternat. Med..

[65] Liang S., Yin Y., Zhang Z., Fang Y., Lu G., Li H., Yin Y., Shen M. (2024). Moxibustion Prevents Tripterygium Glycoside-Induced Oligoasthenoteratozoospermia in Rats via Reduced Oxidative Stress and Modulation of the Nrf2/HO-1 Signaling Pathway. Aging.

[66] Demir S., Mentese A., Usta Z.T., Alemdar N.T., Demir E.A., Aliyazicioglu Y. (2024). Alpha-Pinene Neutralizes Cisplatin-Induced Reproductive Toxicity in Male Rats through Activation of Nrf2 Pathway. Int. Urol. Nephrol..

[67] Demir E.A. (2024). Syringic Acid Alleviates Cisplatin-Induced Ovarian Injury through Modulating Endoplasmic Reticulum Stress, Inflammation and Nrf2 Pathway. J. Trace Elem. Med. Biol..

[68] Lu C.S., Wu C.Y., Wang Y.H., Hu Q.Q., Sun R.Y., Pan M.J., Lu X.Y., Zhu T., Luo S., Yang H.J. (2024). The Protective Effects of Icariin against Testicular Dysfunction in Type 1 Diabetic Mice via AMPK-Mediated Nrf2 Activation and NF-κB p65 Inhibition. Phytomedicine.

[69] Abdelmonem M., Ali S.O., Al-Mokaddem A.K., Ghaiad H.R. (2024). Ameliorating Diabetes-Induced Testicular Dysfunction by Modulating PKC/Nrf2/Bcl-2 Signaling: Protective Role of Sulbutiamine. BioFactors.

[70] Akaras N., Gür C., Caglayan C., Kandemir F.M. (2024). Protective Effects of Naringin against Oxaliplatin-Induced Testicular Damage in Rats: Involvement of Oxidative Stress, Inflammation, Endoplasmic Reticulum Stress, Apoptosis, and Histopathology. Iran. J. Basic Med. Sci..

[71] Ajibare A.J., Akintoye O.O., Folawiyo M.A., Babalola K.T., Omotuyi O.I., Oladun B.T., Aransiola K.T., Odetayo A.F., Olayaki L.A. (2024). Therapeutic Potential of Virgin Coconut Oil in Mitigating Sodium Benzoate-Model of Male Infertility: Role of Nrf2/Hmox-1/NF-kB Signaling Pathway. Iran. J. Basic Med. Sci..

[72] Oyovwi O.M., Ben-Azu B., Tesi E.P., Emojevwe V., Rotu R.A., Moke G.E., Umukoro E., Asiwe J.N., Nwangwa K.E. (2024). Possible Mechanisms Involved in the Protective Effect of Lutein against Cyclosporine-Induced Testicular Damage in Rats. Heliyon.

[73] Wei L., Li S., Ma Y., Ye S., Yuan Y., Zeng Y., Raza T., Xiao F. (2024). Curcumin Attenuates Diphenyl Phosphate-Induced Apoptosis in GC-2spd (ts) Cells through Activated Autophagy via the Nrf2/P53 Pathway. Environ. Toxicol..

[74] Demir E.A., Mentese A., Yilmaz Z.S., Alemdar N.T., Demir S., Aliyazicioglu Y. (2023). Evaluation of the Therapeutic Effects of Arbutin on Cisplatin-Induced Ovarian Toxicity in Rats through Endoplasmic Reticulum Stress and Nrf2 Pathway. Reprod. Biol..

[75] Xi H., Hu Z., Han S., Liu X., Wang L., Hu J. (2023). FSH-Inhibited Autophagy Protects Against Oxidative Stress in Goat Sertoli Cells through p62-Nrf2 Pathway. Theriogenology.

[76] Xu B., He T., Yang H., Dai W., Liu L., Ma X., Ma J., Yang G., Si R., Du X. (2023). Activation of the p62-Keap1-Nrf2 Pathway Protects against Oxidative Stress and Excessive Autophagy in Ovarian Granulosa Cells to Attenuate DEHP-Induced Ovarian Impairment in Mice. Ecotoxicol. Environ. Saf..

[77] Mancuso F., Arato I., Bellucci C., Eugeni E., Stabile A.M., Pistilli A., Brancorsini S., Gaggia F., Calvitti M., Baroni T. (2023). Zinc Restores Functionality in Porcine Prepubertal Sertoli Cells Exposed to Subtoxic Cadmium Concentration via Regulating the Nrf2 Signaling Pathway. Front. Endocrinol..

[78] Qin Z., Song J., Huang J., Jiang S., Zhang G., Huang M., Huang Z., Jin J. (2023). Mitigation of Triptolide-Induced Testicular Sertoli Cell Damage by Melatonin via Regulating the Crosstalk Between SIRT1 and NRF2. Phytomedicine.

[79] Somade O.T., Ajiboye B.O., Osukoya O.A., Jarikre T.A., Oyinloye B.E. (2023). Syringic Acid Ameliorates Testicular Oxidative Stress via the Conservation of Endogenous Antioxidant Markers and Inhibition of the Activated Nrf2-Keap1-NQO1-HO1 Signaling in Methyl Cellosolve-Administered Rats. Pharmacol. Res.-Mod. Chin. Med..

[80] Akanji O.D., Hassanzadeh G., Malekzadeh M., Khanmohammadi N., Khanezad M., Sadeghiani G., Rastegar T. (2023). Pentoxifylline Promotes Spermatogenesis via Upregulation of the Nrf2-ARE Signalling Pathway in a Mouse Model of Germ-Cell Apoptosis Induced by Testicular Torsion–Detorsion. Reprod. Fertil. Dev..

[81] Shati A.A., Khalil M.A. (2023). Acylated Ghrelin Suppresses Doxorubicin-Induced Testicular Damage and Improves Sperm Parameters in Rats via Activation of Nrf2 and Mammalian Target of Rapamycin. J. Cancer Res. Ther..

[82] AlTamimi J.Z., AlFaris N.A., Alshammari G.M., Alagal R.I., Aljabryn D.H., Yahya M.A. (2023). Esculeoside A Alleviates Reproductive Toxicity in Streptozotocin-Diabetic Rats’s Model by Activating Nrf2 Signaling. Saudi J. Biol. Sci..

[83] Adeyi O.E., Somade O.T., James A.S., Adeyi A.O., Ogbonna-Eze S.N., Salako O.Q., Makinde T.V., Ajadi O.M., Nosiru S.A. (2023). Ferulic Acid Mitigates 2-Methoxyethanol-Induced Testicular Oxidative Stress via Combined Downregulation of FoxO1, PTEN, and Modulation of Nrf2-Hmox1-NQO1 Signaling Pathway in Rats. Pharmacol. Res.-Mod. Chin. Med..

[84] Gür F., Cengiz M., Gür B., Cengiz O., Sarıcıçek O., Ayhancı A. (2023). Therapeutic Role of Boron on Acrylamide-Induced Nephrotoxicity, Cardiotoxicity, Neurotoxicity, and Testicular Toxicity in Rats: Effects on Nrf2/Keap-1 Signaling Pathway and Oxidative Stress. J. Trace Elem. Med. Biol..

[85] Akarsu S.A., Gür C., İleritürk M., Akaras N., Küçükler S., Kandemir F.M. (2023). Effect of Syringic Acid on Oxidative Stress, Autophagy, Apoptosis, Inflammation Pathways Against Testicular Damage Induced by Lead Acetate. J. Trace Elem. Med. Biol..

[86] Guan F., Zhang S., Fan L., Sun Y., Ma Y., Cao C., Zhang Y., He M., Du H. (2023). Kunling Wan Improves Oocyte Quality by Regulating the PKC/Keap1/Nrf2 Pathway to Inhibit Oxidative Damage Caused by Repeated Controlled Ovarian Hyperstimulation. J. Ethnopharmacol..

[87] Cai M., Wang J., Sun H., Guo Q., Zhang C., Yao H., Zhao C., Jia Y., Zhu H. (2023). Resveratrol Attenuates Hydrogen Peroxide-Induced Injury of Rat Ovarian Granulosa-Lutein Cells by Resisting Oxidative Stress via the SIRT1/Nrf2/ARE Signaling Pathway. Curr. Pharm. Des..

[88] Qian Z., Li C., Zhao W., He Z., Xue M., Wang S., Cheng X., Ma R., Ge X. (2023). Chronic Oral Exposure to Short Chain Chlorinated Paraffins Induced Testicular Toxicity by Promoting NRF2-Mediated Oxidative Stress. Toxicol. Lett..

[89] Niu C., Jiang D., Guo Y., Wang Z., Sun Q., Wang X., Ling W., An X., Ji C., Li S. (2023). Spermidine Suppresses Oxidative Stress and Ferroptosis by Nrf2/HO-1/GPX4 and Akt/FHC/ACSL4 Pathway to Alleviate Ovarian Damage. Life Sci..

[90] Liu M., Zhou X., Wang X.J., Wang Y.S., Yang S.J., Ding Z.M., Zhang S.X., Zhang L.D., Duan Z.Q., Liang A.X. (2023). Curcumin Alleviates Bisphenol AF-Induced Oxidative Stress and Apoptosis in Caprine Endometrial Epithelial Cells via the Nrf2 Signaling Pathway. Environ. Toxicol..

[91] Feng Z., Wang T., Sun Y., Chen S., Hao H., Du W., Zou H., Yu D., Zhu H., Pang Y. (2023). Sulforaphane Suppresses Paraquat-Induced Oxidative Damage in Bovine In Vitro-Matured Oocytes through Nrf2 Transduction Pathway. Ecotoxicol. Environ. Saf..

[92] Arab H.H., Fikry E.M., Alsufyani S.E., Ashour A.M., El-Sheikh A.A., Darwish H.W., Al-Hossaini A.M., Saad M.A., Al-Shorbagy M.Y., Eid A.H. (2023). Stimulation of Autophagy by Dapagliflozin Mitigates Cadmium-Induced Testicular Dysfunction in Rats: The Role of AMPK/mTOR and SIRT1/Nrf2/HO-1 Pathways. Pharmaceuticals.

[93] Odetayo A.F., Adeyemi W.J. (2023). Omega-3 Fatty Acid Ameliorates Bisphenol F-Induced Testicular Toxicity by Modulating Nrf2/NFkB Pathway and Apoptotic Signaling. Front. Endocrinol..

[94] Liang Y., Lu J., Yi W., Cai M., Shi W., Li B., Zhang Z., Jiang F. (2023). 1α, 25-Dihydroxyvitamin D3 Supplementation Alleviates Perfluorooctanesulfonate Acid-Induced Reproductive Injury in Male Mice: Modulation of Nrf2 Mediated Oxidative Stress Response. Environ. Toxicol..

[95] Habiba E.S., Harby S.A., El-Sayed N.S., Omar E.M., Bakr B.A., Augustyniak M., El-Samad L.M., Hassan M.A. (2023). Sericin and Melatonin Mitigate Diethylnitrosamine-Instigated Testicular Impairment in Mice: Implications of Oxidative Stress, Spermatogenesis, Steroidogenesis, and Modulation of Nrf2/WT1/SF-1 Signaling Pathways. Life Sci..

[96] Huang W., Cao Z., Cui Y., Huo S., Shao B., Song M., Cheng P., Li Y. (2023). Lycopene Ameliorates Aflatoxin B1-Induced Testicular Lesion by Attenuating Oxidative Stress and Mitochondrial Damage with Nrf2 Activation in Mice. Ecotoxicol. Environ. Saf..

[97] Heidarizadi S., Rashidi Z., Jalili C., Mansouri K., Rashidi I., Mahaki B., Gholami M. (2023). Melatonin Protects Mouse Type A Spermatogonial Stem Cells against Oxidative Stress via The Mitochondrial Thioredoxin System. Cell J..

[98] Sarawi W.S., Alhusaini A.M., Fadda L.M., Alomar H.A., Albaker A.B., Alghibiwi H.K., Aljrboa A.S., Alotaibi A.M., Hasan I.H., Mahmoud A.M. (2022). Nano-Curcumin Prevents Copper Reproductive Toxicity by Attenuating Oxidative Stress and Inflammation and Improving Nrf2/HO-1 Signaling and Pituitary-Gonadal Axis in Male Rats. Toxics.

[99] Bakr A.G., Hassanein E.H., Ali F.E., El-Shoura E.A. (2022). Combined Apocynin and Carvedilol Protect against Cadmium-Induced Testicular Damage via Modulation of Inflammatory Response and Redox-Sensitive Pathways. Life Sci..

[100] Bartolini D., Arato I., Mancuso F., Giustarini D., Bellucci C., Vacca C., Aglietti M.C., Stabile A.M., Rossi R., Cruciani G. (2022). Melatonin Modulates Nrf2 Activity to Protect Porcine Pre-Pubertal Sertoli Cells from the Abnormal H2O2 Generation and Reductive Stress Effects of Cadmium. J. Pineal Res..

[101] Fan L., Guan F., Ma Y., Zhang Y., Li L., Sun Y., Cao C., Du H., He M. (2022). N-Acetylcysteine Improves Oocyte Quality through Modulating the Nrf2 Signaling Pathway to Ameliorate Oxidative Stress Caused by Repeated Controlled Ovarian Hyperstimulation. Reprod. Fertil. Dev..

[102] Zhang J., Fang Y., Tang D., Xu X., Zhu X., Wu S., Yu H., Cheng H., Luo T., Shen Q. (2022). Activation of MT1/MT2 to Protect Testes and Leydig Cells against Cisplatin-Induced Oxidative Stress through the SIRT1/Nrf2 Signaling Pathway. Cells.

[103] Lin X., Zhu L., Gao X., Kong L., Huang Y., Zhao H., Chen Y., Wen L., Li R., Wu J. (2022). Ameliorative Effect of Betulinic Acid against Zearalenone Exposure Triggers Testicular Dysfunction and Oxidative Stress in Mice via p38/ERK MAPK Inhibition and Nrf2-Mediated Antioxidant Defense Activation. Ecotoxicol. Environ. Saf..

[104] Yan R., Wang H., Zhu J., Wang T., Nepovimova E., Long M., Li P., Kuca K., Wu W. (2022). Procyanidins Inhibit Zearalenone-Induced Apoptosis and Oxidative Stress of Porcine Testis Cells through Activation of Nrf2 Signaling Pathway. Food Chem. Toxicol..

[105] Chen H., Chen J., Shi X., Li L., Xu S. (2022). Naringenin Protects Swine Testis Cells from Bisphenol A-Induced Apoptosis via Keap1/Nrf2 Signaling Pathway. BioFactors.

[106] Hemati U., Moshajari M., Jalali Mashayekhi F., Bayat M., Moslemi A., Baazm M. (2022). The Effect of Curcumin on NRF2/Keap1 Signalling Pathway in the Epididymis of Mouse Experimental Cryptorchidism. Andrologia.

[107] Ouyang H., Zhu H., Li J., Chen L., Zhang R., Fu Q., Li X., Cao C. (2022). Fumonisin B1 Promotes Germ Cells Apoptosis Associated with Oxidative Stress-Related Nrf2 Signaling in Mice Testes. Chem.-Biol. Interact..

[108] Chen Z., Zuo Z., Chen K., Yang Z., Wang F., Fang J., Cui H., Guo H., Ouyang P., Chen Z. (2022). Activated Nrf-2 Pathway by Vitamin E to Attenuate Testicular Injuries of Rats with Sub-Chronic Cadmium Exposure. Biol. Trace Elem. Res..

[109] Wang Y., Li J., Gu J., He W., Ma B., Fan H. (2022). Hyperoside, A Natural Flavonoid Compound, Attenuates Triptolide-Induced Testicular Damage by Activating the Keap1-Nrf2 and SIRT1-PGC1α Signalling Pathway. J. Pharm. Pharmacol..

[110] Shahidi M., Moradi A., Dayati P. (2022). Zingerone Attenuates Zearalenone-Induced Steroidogenesis Impairment and Apoptosis in TM3 Leydig Cell Line. Toxicon.

[111] Abdelzaher W.Y., Mostafa-Hedeab G., Sayed AboBakr Ali A.H., Fawzy M.A., Ahmed A.F., Bahaa El-deen M.A., Welson N.N., Aly Labib D.A. (2022). Idebenone Regulates Sirt1/Nrf2/TNF-α Pathway with Inhibition of Oxidative Stress, Inflammation, and Apoptosis in Testicular Torsion/Detorsion in Juvenile Rats. Hum. Exp. Toxicol..

[112] Ukwenya V.O., Aderemi A.S., Alese O.M., Augustine O.A. (2022). Caffeic Acid Abrogates Amyloidosis, Hypospermatogenesis and Cell Membrane Alterations in the Testes and Epididymis of Fructose-Diabetic Rats by Upregulating Steroidogenesis, PCNA and Nrf2 Expression. Tissue Cell.

[113] Adeniran S.O., Zheng P., Feng R., Adegoke E.O., Huang F., Ma M., Wang Z., Ifarajimi O.O., Li X., Zhang G. (2022). The Antioxidant Role of Selenium via GPx1 and GPx4 in LPS-Induced Oxidative Stress in Bovine Endometrial Cells. Biol. Trace Elem. Res..

[114] Cao L., Zhao J., Ma L., Chen J., Xu J., Rahman S.U., Feng S., Li Y., Wu J., Wang X. (2021). Lycopene Attenuates Zearalenone-Induced Oxidative Damage of Piglet Sertoli Cells through the Nuclear Factor Erythroid-2 Related Factor 2 Signaling Pathway. Ecotoxicol. Environ. Saf..

[115] Li G., Zhang P., Ma Z. (2021). Qiangjing Tablets Regulate Apoptosis and Oxidative Stress via Keap/Nrf2 Pathway to Improve Reproductive Function in Asthenospermia Rats. Front. Pharmacol..

[116] Jia L.I., Yu-Hang C.H., Jia-Yu X.U., Jiang-Ying L.I., Jia-Cheng F.U., Xiu-Ping C.A., Huang J., Zheng Y.H. (2021). Effects of Chitooligosaccharide-Zinc on the Ovarian Function of Mice with Premature Ovarian Failure via the SESN2/NRF2 Signaling Pathway. Chin. J. Nat. Med..

[117] Al-Megrin W.A., Alomar S., Alkhuriji A.F., Metwally D.M., Mohamed S.K., Kassab R.B., Abdel Moneim A.E., El-Khadragy M.F. (2020). Luteolin Protects against Testicular Injury Induced by Lead Acetate by Activating the Nrf2/HO-1 Pathway. IUBMB Life.

[118] Yuan L., Li Q., Bai D., Shang X., Hu F., Chen Z., An T., Chen Y., Zhang X. (2020). La_2_O_3_ Nanoparticles Induce Reproductive Toxicity Mediated by the Nrf-2/ARE Signaling Pathway in Kunming Mice. Int. J. Nanomed..

[119] Kassab R.B., Lokman M.S., Daabo H.M., Gaber D.A., Habotta O.A., Hafez M.M., Zhery A.S., Moneim A.E., Fouda M.S. (2020). Ferulic Acid Influences Nrf2 Activation to Restore Testicular Tissue from Cadmium-Induced Oxidative Challenge, Inflammation, and Apoptosis in Rats. J. Food Biochem..

[120] Abdel-Wahab B.A., Alkahtani S.A., Elagab E.A. (2020). Tadalafil Alleviates Cisplatin-Induced Reproductive Toxicity through the Activation of the Nrf2/HO-1 Pathway and the Inhibition of Oxidative Stress and Apoptosis in Male Rats. Reprod. Toxicol..

[121] Khadrawy O., Gebremedhn S., Salilew-Wondim D., Rings F., Neuhoff C., Hoelker M., Schellander K., Tesfaye D. (2020). Quercetin Supports Bovine Preimplantation Embryo Development under Oxidative Stress Condition via Activation of the Nrf2 Signalling Pathway. Reprod. Domest. Anim..

[122] Güvenç M., Cellat M., Gökçek İ., Arkalı G., Uyar A., Tekeli İ.O., Yavaş İ. (2020). Tyrosol Prevents AlCl3 Induced Male Reproductive Damage by Suppressing Apoptosis and Activating the Nrf-2/HO-1 Pathway. Andrologia.

[123] Chen S., Yang S., Wang M., Chen J., Huang S., Wei Z., Cheng Z., Wang H., Long M., Li P. (2020). Curcumin Inhibits Zearalenone-Induced Apoptosis and Oxidative Stress in Leydig Cells via Modulation of the PTEN/Nrf2/Bip Signaling Pathway. Food Chem. Toxicol..

[124] Yang S.H., Li P., Yu L.H., Li L., Long M., Liu M.D., He J.B. (2019). Sulforaphane Protects against Cadmium-Induced Oxidative Damage in Mouse Leydig Cells by Activating Nrf2/ARE Signaling Pathway. Int. J. Mol. Sci..

[125] Renu K., Gopalakrishnan A.V. (2019). Deciphering the Molecular Mechanism during Doxorubicin-Mediated Oxidative Stress, Apoptosis through Nrf2 and PGC-1α in a Rat Testicular Milieu. Reprod. Biol..

[126] Zhao Y., Li M.Z., Shen Y., Lin J., Wang H.R., Talukder M., Li J.L. (2019). Lycopene Prevents DEHP-Induced Leydig Cell Damage with the Nrf2 Antioxidant Signaling Pathway in Mice. J. Agric. Food Chem..

[127] Hu Y., Wang Y., Yan T., Feng D., Ba Y., Zhang H., Zhu J., Cheng X., Cui L., Huang H. (2019). N-Acetylcysteine Alleviates Fluoride-Induced Testicular Apoptosis by Modulating IRE1α/JNK Signaling and Nuclear Nrf2 Activation. Reprod. Toxicol..

[128] Yang S.H., He J.B., Yu L.H., Li L., Long M., Liu M.D., Li P. (2019). Protective Role of Curcumin in Cadmium-Induced Testicular Injury in Mice by Attenuating Oxidative Stress via Nrf2/ARE Pathway. Environ. Sci. Pollut. Res..

[129] Shi X., Fu L. (2019). Piceatannol Inhibits Oxidative Stress through Modification of Nrf2-Signaling Pathway in Testes and Attenuates Spermatogenesis and Steroidogenesis in Rats Exposed to Cadmium during Adulthood. Drug Des. Devel. Ther..

[130] Yang S.H., Yu L.H., Li L., Guo Y., Zhang Y., Long M., Li P., He J.B. (2018). Protective Mechanism of Sulforaphane on Cadmium-Induced Sertoli Cell Injury in Mice Testis via Nrf2/ARE Signaling Pathway. Molecules.

[131] He L., Li P., Yu L.H., Li L., Zhang Y., Guo Y., Long M., He J.B., Yang S.H. (2018). Protective Effects of Proanthocyanidins against Cadmium-Induced Testicular Injury through the Modification of Nrf2-Keap1 Signal Path in Rats. Environ. Toxicol. Pharmacol..

[132] Chen X., Song Q.L., Li Z.H., Ji R., Wang J.Y., Cao M.L., Mu X.F., Zhang Y., Guo D.Y., Yang J. (2023). Pterostilbene Ameliorates Oxidative Damage and Ferroptosis in Human Ovarian Granulosa Cells by Regulating the Nrf2/HO-1 Pathway. Arch. Biochem. Biophys..

[133] Taheri M., Roudbari N.H., Amidi F., Parivar K. (2022). Investigating the Effect of Sulforaphane on AMPK/AKT/NRF2 Pathway in Human Granulosa-Lutein Cells under H_2_O_2_-Induced Oxidative Stress. Eur. J. Obstet. Gynecol. Reprod. Biol..

[134] Ma Y., Hao G., Lin X., Zhao Z., Yang A., Cao Y., Zhang S., Fan L., Geng J., Zhang Y. (2022). Morroniside Protects Human Granulosa Cells against H_2_O_2_-Induced Oxidative Damage by Regulating the Nrf2 and MAPK Signaling Pathways. Evid. Based Complement. Alternat. Med..

[135] Afzali A., Amidi F., Koruji M., Nazari H., Gilani M.A., Sanjbad A.S. (2022). Astaxanthin Relieves Busulfan-Induced Oxidative Apoptosis in Cultured Human Spermatogonial Stem Cells by Activating the Nrf-2/HO-1 Pathway. Reprod. Sci..

[136] Taheri M., Roudbari N.H., Amidi F., Parivar K. (2021). The Protective Effect of Sulforaphane against Oxidative Stress in Granulosa Cells of Patients with Polycystic Ovary Syndrome (PCOS) through Activation of AMPK/AKT/NRF2 Signaling Pathway. Reprod. Biol..

[137] Sammad A., Luo H., Hu L., Zhu H., Wang Y. (2022). Transcriptome Reveals Granulosa Cells Coping through Redox, Inflammatory and Metabolic Mechanisms under Acute Heat Stress. Cells.

[138] Wang Y., Yang C., Nahla Abdalla Hassan E., Li C., Yang F., Wang G., Li L. (2019). HO-1 Reduces Heat Stress-Induced Apoptosis in Bovine Granulosa Cells by Suppressing Oxidative Stress. Aging.

[139] Deng C.C., Zhang J.P., Huo Y.N., Xue H.Y., Wang W., Zhang J.J., Wang X.Z. (2022). Melatonin Alleviates the Heat Stress-Induced Impairment of Sertoli Cells by Reprogramming Glucose Metabolism. J. Pineal Res..

[140] Toosinia S., Davoodian N., Arabi M., Kadivar A. (2024). Ameliorating Effect of Sodium Selenite on Developmental and Molecular Response of Bovine Cumulus-Oocyte Complexes Matured In vitro Under Heat Stress Condition. Biol. Trace Elem. Res..

[141] Ho K.T., Homma K., Takanari J., Bai H., Kawahara M., Nguyen K.T., Takahashi M. (2021). Standardized Extract of Asparagus officinalis Stem Improves HSP70-Mediated Redox Balance and Cell Functions in Bovine Cumulus-Granulosa Cells. Sci. Rep..

[142] Badr G., Abdel-Tawab H.S., Ramadan N.K., Ahmed S.F., Mahmoud M.H. (2018). Protective Effects of Camel Whey Protein against Scrotal Heat-Mediated Damage and Infertility in the Mouse Testis through YAP/Nrf2 and PPAR-Gamma Signaling Pathways. Mol. Reprod. Dev..

[143] Kumar S.S., Manna K., Das A. (2018). Tender Coconut Water Attenuates Heat Stress-Induced Testicular Damage through Modulation of the NF-κB and Nrf2 Pathways. Food Funct..

